# Effect of hydrogen on semiconductive properties of passive film on ferrite and austenite phases in a duplex stainless steel

**DOI:** 10.1038/s41598-017-03480-8

**Published:** 2017-06-12

**Authors:** L. Q. Guo, S. X. Qin, B. J. Yang, D. Liang, L. J. Qiao

**Affiliations:** Corrosion and Protection Center, Key Laboratory for Environmental Fracture (MOE), University of Science and Technology Beijing, Beijing, 100083 People’s Republic of China

## Abstract

Hydrogen effect on semiconductivity and compositions of passive films formed on ferrite and austenite phases in a duplex stainless steel were investigated by current sensing atomic force microscopy and X-ray photoelectron spectroscopy. It is demonstrated that hydrogen significantly increases the conductivity of passive film due to the increase of OH^−^/O^2−^ ratio. The passive film on austenite has higher conductivity than that on ferrite after hydrogen charging due to more hydrogen in austenite. The presence of hydrogen causes an inversion of conductivity type of passive film from p-type to n-type, attributed to the chemical composition change.

## Introduction

The high corrosion resistance of stainless steels is mainly due to the surface passive films. Their semiconductive properties play an important role in the film breakdown mechanism^1–5^. However, the generation and the adsorption of hydrogen are unavoidable in many processes such as heat treatment, pickling and cathodic protection, which influences the electrochemical behavior of metallic materials. Considerable efforts have been focused on the hydrogen effect on the electronic properties of passive films^6–10^. It has shown that hydrogen increases the donor density and surface area activity of the passive films, and deteriorated the passive film stability^8–10^. However, the role of hydrogen in relation to the semiconductive properties of passive films in stainless steel remains far from clarity. Moreover, there is no information available on the semiconducting behavior of hydrogen-containing passive films on duplex stainless steel. This is mainly because duplex stainless steel has a heterogeneous structure composed of ferrite and austenite, leading to the differences in semiconductivity of passive films between the two phases.

The classical Mott-Schottky analysis using electrochemical impedance spectroscopy and photo-electrochemical methods have been used to identify semiconductor characteristics of passive films, including semiconductor type. However, these are relatively macroscopic measuring methods, meaning that the difference in semiconductive properties between ferrite and austenite can not be identified. Also, the electrochemical impedance spectroscopy test is carried out in the solution and the results depend on the electrolytic media and the applied potential^11–13^, and thus causing uncertainty of measurement results. It is necessary to investigate the hydrogen-induced change of passive film semiconductive properties by precise and spatially resolved measurements.

Current sensing atomic force microscope (CSAFM) is an effective method to investigate the hydrogen-induced local semiconductive properties’ change of passive film on duplex stainless steel. It is because that CSAFM can acquire the electrical conductivity of the passive film covering the ferrite and austenite phases, respectively^14–16^. Moreover, local current-voltage (I–V) curves can be obtained to identify the corresponding semiconductor type of passive film in air environment at the microscopic scale, which is independent of the measurement conditions^16, 17^. CSAFM can provide valuable information concerning the passive film properties at a microscopic level to help understanding the mechanisms of passivity breakdown. We used CSAFM to characterize the semiconductivity of passive films on ferrite and austenite phases before and after passivation at various potentials^16^.

The present work aims to characterize the hydrogen-induced changes of semiconductivity of passive films formed on ferrite and austenite phases in duplex stainless steel through current mapping and I–V curves with CSAFM. The change of the chemical composition of the passive films before and after hydrogen charging was characterized by X-ray photoelectron spectroscopy (XPS) to confirm CSAFM measurement results.

## Results and Discussion

**Figure 1:**
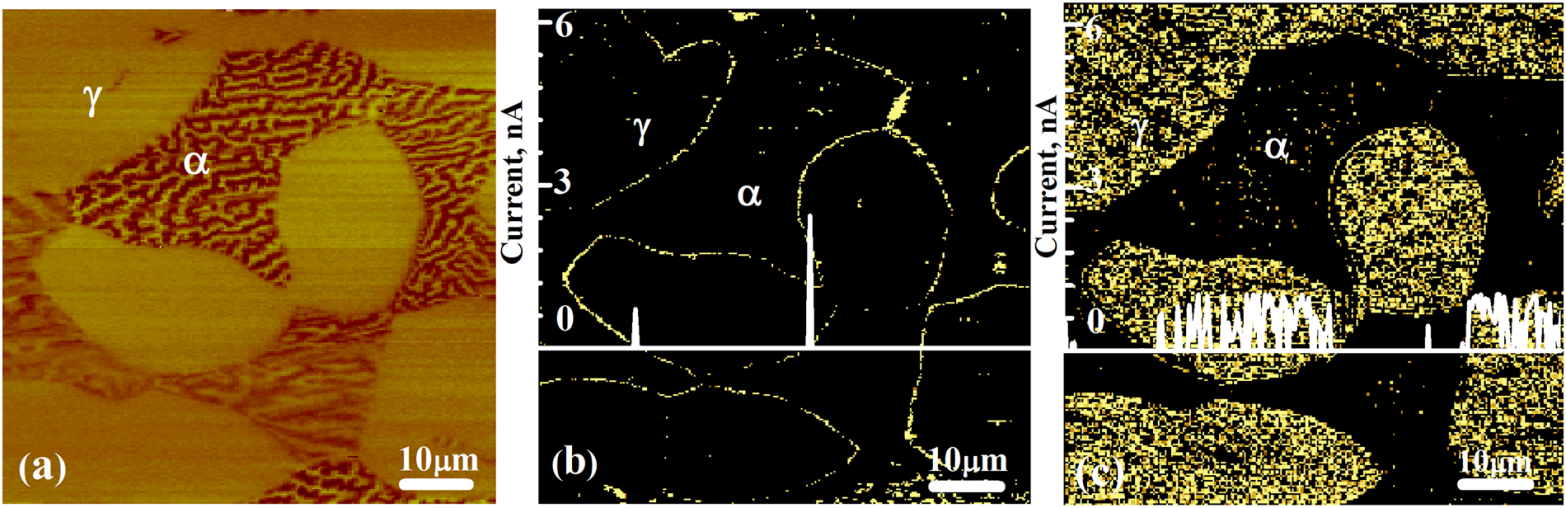
(**a**) MFM image before hydrogen charging, and corresponding conductive maps (in nA) with line current profiles for the passive films without hydrogen (**b**) and with hydrogen (**c**) on the same region as MFM image.

For AFM analysis, MFM (magnetic force microscopy) was employed to identify the ferrite and austenite phases, thanks to their distinctive magnetic characteristics. A MFM image is illustrated in Fig. 1(a). As shown, the ferrite phase has a striped appearance due to its ferromagnetic behavior, while the paramagnetic austenite phase shows a uniform appearance.

Figure 1(b,c) present CSAFM maps with 1 V applied tip bias, along with the corresponding current profiles of the passive films formed before and after hydrogen charging in the same area as MFM image. On the uncharged specimen (Fig. 1(b)), the passive films on both ferrite and austenite phases exhibit lower conductivity (darker color), meaning that there are stable passive films on the uncharged specimen^16^. The average current of passive films on the austenite and ferrite is about 0.09 nA and 0.05 nA, respectively. It is expected that the passive films on the boundaries of ferrite and austenite phases show higher conductivity (bright color), the reason being that the boundaries include an enormous number of lattice mismatches, i.e. defects which leads to formation of a defective film. However, after hydrogen charging, the current of passive films increased significantly on both phases, especially on austenite shown in Fig. 1(c). The average current of passive film on the austenite and ferrite is about 1 nA and 0.1 nA, respectively. The current increased over 11 times in austenite and 2 times in ferrite after hydrogen charging. This result illustrates that hydrogen causes a remarkably increase of electrical conductivity of passive film, namely hydrogen makes the passive film more active, which is consistent with reports^6–9^. Additionally, the austenite has a much higher current than the ferrite after hydrogen charging, and the current of austenite is about 10 times higher than that of ferrite. It is because there is much more hydrogen in the austenite than that in ferrite (hydrogen has a higher solubility and a lower diffusivity in the face-centered cubic austenite phase, as compared with in the body-centered cubic ferrite phase)^18, 19^.

**Figure 2:**
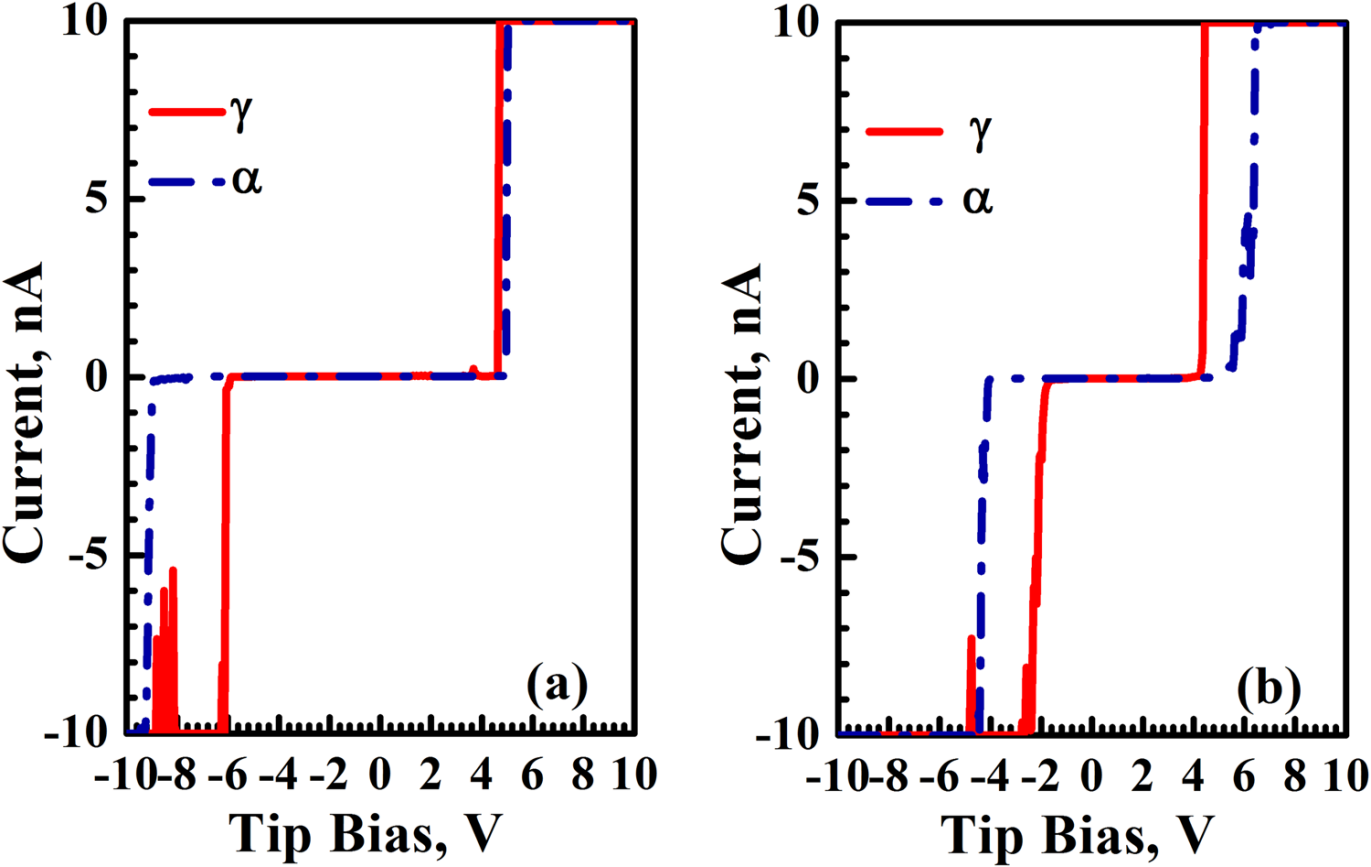
Typical I–V curves obtained from the passive films on the ferrite and austenite phases formed on duplex stainless steel (**a**) before hydrogen charging and (**b**) after hydrogen charging.

Figure 2 exhibits the I–V curves obtained on the passive films before and after hydrogen charging covering the ferrite and austenite phases. The zero-current region on I–V curve corresponds to the band gap energy width of the overlayer^14^. It can be seen that after hydrogen charging, the width of the zero-current region on both phases becomes smaller, and the width on austenite is much smaller than that on ferrite. The I–V curves demonstrate that passive films on the hydrogen-charged specimen have a higher conductivity than uncharged one, and the austenite has much higher conductivity than the ferrite after hydrogen charging. This is consistent with the current maps shown in Fig. 1.

According to the method of determining the passive film semiconductor type using I–V curves in our previous works^16, 17^ (if measured asymmetric I–V curve has higher resistance with positive applied voltage, the semiconductor is regarded as the n-type, otherwise the semiconductor is thought to be the p-type), the semiconductor type can be identified. The passive films formed on both ferrite and austenite before hydrogen charging appear as p-type semiconductors, but the passive films formed on both phases after hydrogen charging exhibit n-type semiconductor. It is well known that the passive films exhibit n-type or p-type semiconducting behavior, which is related to their chemical composition^20^. The passive films of stainless steels consist primarily of chromium and iron oxides or hydroxides^20^. Generally, Cr_2_O_3_, FeO, Cr(OH)_3_ behave as the p-type semiconductor, while Fe_2_O_3_, FeOOH, CrO_3_, Fe_3_O_4_ exhibit n-type semiconductor properties^2, 21–24^.

**Figure 3:**
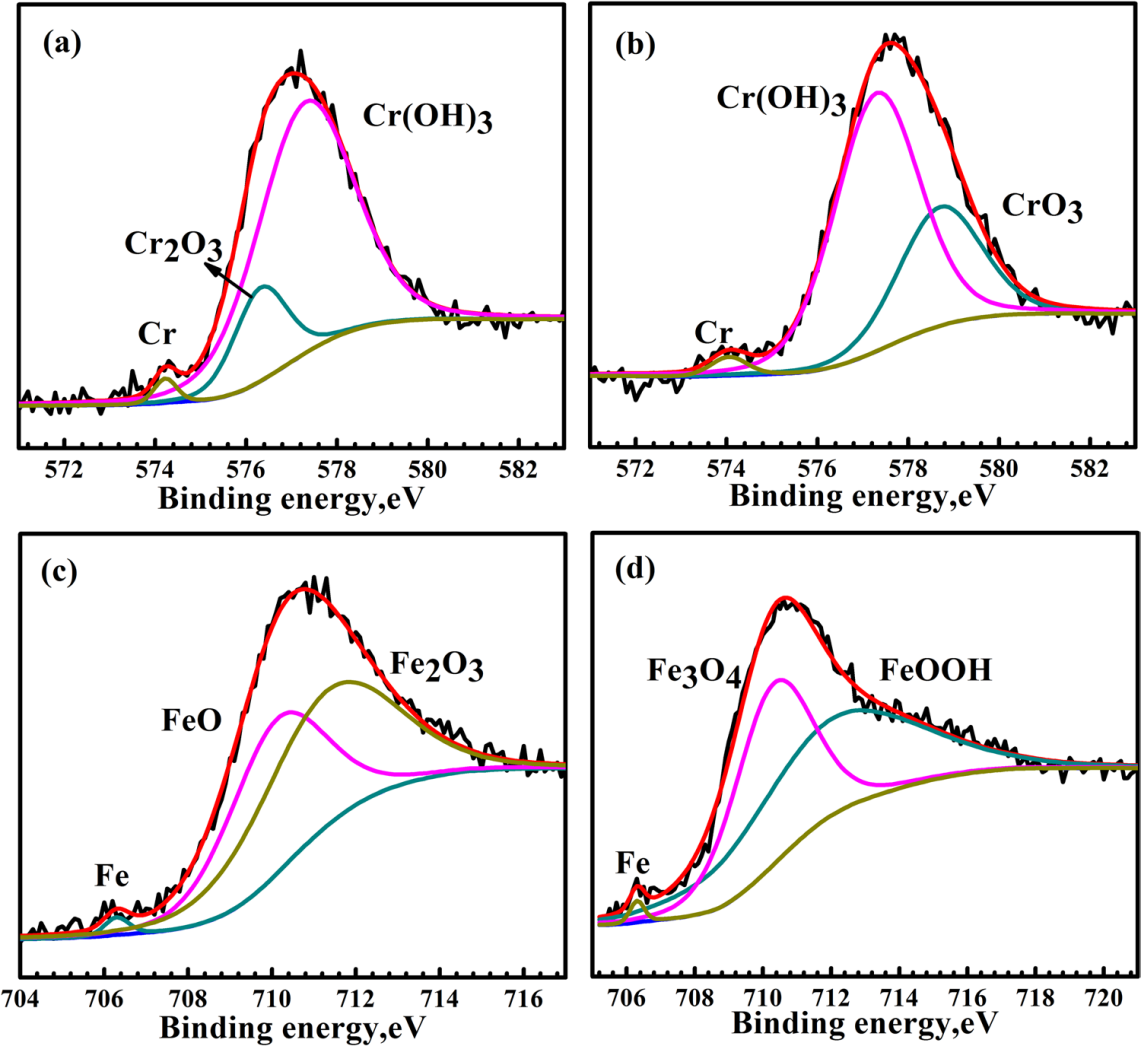
XPS spectra of Cr 2p_3/2_ and Fe 2p_3/2_ of the passive films formed on the uncharged surface (**a**,**c**) and hydrogen-charged surface (**b**,**d**).

To further confirm the CSAFM measurement result that hydrogen induced change of conductivity type of passive films, XPS analysis was carried out to examine the change in the film surface composition caused by hydrogen charging. Figure 3 shows the metallic and oxidized states of Cr 2p_3/2_ and Fe 2p_3/2_ of the passive films. Peak signals of Mo and Ni were relatively weak and their XPS spectra are therefore not represented here. The Cr 2p_3/2_ signals in Fig. 3(a) show the presence of the three components for the passive films formed on the uncharged specimen: Cr_(met)_ (574.2 eV), Cr(OH)_3_ (577.3 eV) and Cr_2_O_3_ (576.3 eV). The quantitative evaluation yielded 1.87% of Cr_(met)_, 81.32% of Cr(OH)_3_ and 16.80% of Cr_2_O_3_, thus Cr(OH)_3_ and Cr_2_O_3_ are the primary constituents of the passive films formed on the uncharged specimen. For the spectra of the passive films formed on the hydrogen-charged specimen, in the Cr 2p_3/2_ spectra there exist three constituents representing Cr_(met)_ (574.2 eV), CrO_3_ (578.3 eV), Cr(OH)_3_ (577.3 eV) shown in Fig. 3(b). Compared with passive films on the uncharged specimen, the concentration of Cr_2_O_3_ and Cr(OH)_3_ became less which were 0% and 58.87% respectively, but a new constituent peak appears which is CrO_3_ (39.03%). In the Fe 2p_3/2_ spectra there are three constituent peaks of Fe_(met)_ (706.5 eV), Fe_2_O_3_ (711.5 eV) and FeO (709.9 eV) for the passive films on the uncharged specimen (see Fig. 3(c)). The quantitative evaluation yielded 1.29% of Fe_(met)_, 54.60% of Fe_2_O_3_, and 44.11% of FeO. Compared with passive films on uncharged specimen, there are no FeO and Fe_2_O_3_, but Fe_3_O_4_ (710.4 eV) and FeOOH (712.0 eV) are present in the Fe 2p_3/2_ spectra for the passive films on hydrogen-charged specimen (see Fig. 3(d)), which were 1.38% of Fe_(met)_, 44.91% of Fe_3_O_4_ and 53.71% of FeOOH.

The passive films on the uncharged specimen have Cr(OH)_3_, Cr_2_O_3_ and FeO, leading to the increase of p-type semiconductive properties. The composition of the passive films after hydrogen charging is dramatically changed. Specifically, CrO_3_, FeOOH and Fe_3_O_4_ appeared after hydrogen charging, matching the n-type semiconducting character. The formation of CrO_3_ and Fe_3_O_4_ is probably due to the hydrogen effect on the corrosion of substrate, which needs further investigation. Also, the peaks of the p-type semiconductor oxides such as FeO, Cr_2_O_3_ and Cr(OH)_3_ are remarkably decreased after hydrogen charging.

Table 1 summarizes the chemical composition in the passive films formed before and after hydrogen charging. XPS quantitative analysis reveals that the ratio of the p-type and n-type semiconductor compositions for the passive films after hydrogen charging is remarkably decreased. The ratio for the passive films before and after hydrogen charging is 1.81 and 0.26, respectively. The results confirmed that the presence of hydrogen in duplex stainless steel causes an inversion of semiconductivity type of passive film from p-type towards n-type, which is consistent with the report^6^.

**Table 1:** The elemental composition of the passive film formed on duplex stainless steel before and after hydrogen charging.

Passive film	Composition in wt.%											
	Fe	FeO	Fe_2_O_3_	Fe_3_O_4_	FeOOH	Cr	Cr_2_O_3_	Cr(OH)_3_	CrO_3_	O^2−^	OH^−^	H_2_O
Before hydrogen charging	1.29	44.11	54.60	0	0	1.87	16.80	81.32	0	35.29	58.98	5.73
After hydrogen charging	1.38	0	0	44.91	53.71	2.10	0	58.87	39.03	11.14	75.44	4.65

**Figure 4:**
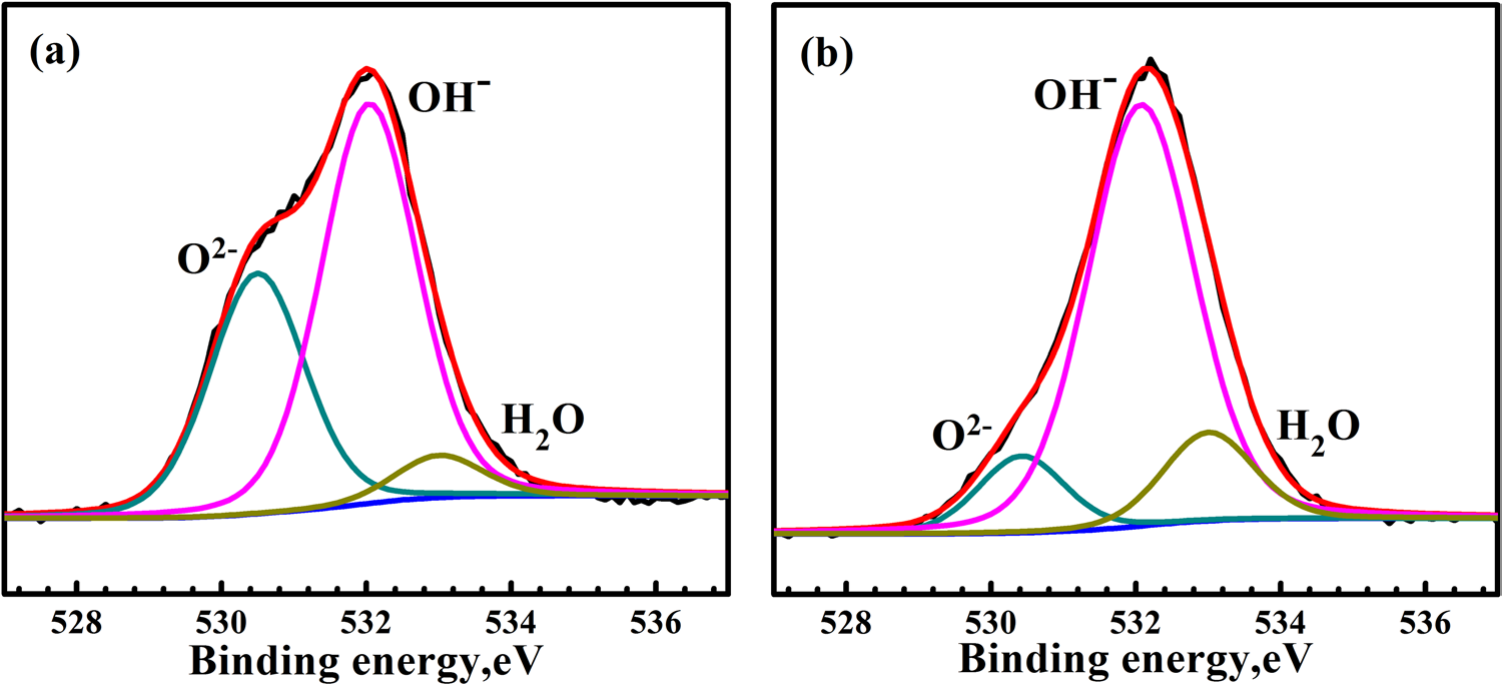
XPS spectra of O 1 s of the passive films formed on the uncharged surface (**a**) and hydrogen-charged surface (**b**).

Figure 4 shows the spectra of O 1 s for the passive films formed on uncharged and hydrogen-charged specimens. The O1s spectrum of passive films on uncharged specimen (see Fig. 4(a)) is composed of three peaks, which are absorbed water at 533.0 eV, O^2−^ species at 530.42 eV, and OH^−^ species at 532.0 eV. The quantitative calculation reveals 5.73% of absorbed water, 35.29% of O^2−^, and 58.98% of OH^−^. The O1s spectrum of passive films on the specimen charged with hydrogen (Fig. 4(b)) is composed of three peaks, which are 4.65% of absorbed water (533.0 eV), 11.14% of O^2−^ (530.42 eV), and 75.44% of OH^−^ (532.0) species (see Table 1). XPS quantitative analysis reveals that the OH^−^/O^2−^ ratio is significantly changed after hydrogen charging, which is 1.67 and 6.77 for the passive films on uncharged and charged specimens respectively. It is indicated that the presence of hydrogen can remarkably increase OH^−^/O^2−^ ratio in the passive film, which matches some reports^6, 7, 25^. Hydroxide has more defects than oxide^8^, thus the conductivity of the passive films formed on hydrogen-charged specimen is better than that formed on uncharged specimen. This matches CSAFM mapping and I–V curves measurements shown in Figs 1 and 2. More hydrogen in the austenite makes the passive films formed on austenite more active than that on ferrite, i.e. the passive films on austenite is more electrically conductive than that on ferrite, as shown in Figs 1 and 2. This matches our previous work that pitting nucleates initially inside the austenite phase in duplex stainless steel after hydrogen charging^26, 27^, meaning that hydrogen could lower pitting corrosion resistance.

## Conclusions

In summary, with local current mapping and I–V curve measurement using CSAFM, we analyzed semiconducting behavior of passive films formed on austenite and ferrite in a duplex stainless steel before and after hydrogen charging. It was demonstrated that the passive films formed on the specimen charged with hydrogen had much higher current, compared to passive films formed on the uncharged specimen. This means that hydrogen increases the conductivity of passive films. This is due to the increase of ratio of OH^−^/O^2−^ in passive films after hydrogen charging, confirmed by XPS analysis. The passive films on the austenite had higher conductivity after hydrogen charging than that on the ferrite due to much more hydrogen in the austenite. I–V curves demonstrated that the presence of hydrogen causes an inversion of conductivity type of passive films from p-type to n-type, attributed to the chemical composition change, supported by XPS results.

## Methods

### Sample preparation

The material under study is a conventional 2507 duplex stainless steel^16, 17, 26, 27^. Specimen cut from the steel was wet ground with SiC paper up to 2000 grit, and then mechanical polished using a 0.5 μm-diamond paste. The sample surface was electrochemically polished in a mixed solution of HNO_3_:H_2_O = 1:1 for 20 sec under an applied voltage of 1.2 V to remove any residual deformation or stress in the surface layers due to mechanical polishing. The specimen was ultrasonically cleaned in ethanol and dried by a N_2_ gas flow.

### Passive film formation

Specimen was initially pretreated cathodically at −0.8 V_SCE_ (voltage per saturated calomel electrode) for 30 min to remove the native oxide film and then polarized in a borate buffer solution containing 0.05 M H_3_BO_4_ + 0.075 M Na_2_B_4_O_7_ (pH = 9.2) for 2 h at 0.1 V_SCE_ chosen based on the polarization curve to form stable passive film. The experiments were carried out at room temperature with the electrochemical cell consisted of specimen as the working electrode, and a saturated calomel and platinum as reference and counter electrodes.

### Cathodic hydrogen charging

Before hydrogen charging, the specimen was pretreated cathodically again to remove the passive film. The hydrogen charging was carried out at room temperature in 0.22 M NaOH solution with an addition of 0.2 g/L thiourea as hydrogen recombination poison to promote hydrogen absorption. In order to avoid hydrogen-induced phase transformation, the charging current density was 1 mA/cm^2^ and the time of hydrogen charging was 48 hours. Then the passive films were formed on the hydrogen charged specimen with same parameters as for the uncharged specimen.

### CSAFM and MFM measurements

CSAFM measurements of passive films before and after hydrogen charging were performed using Agilent 5500 AFM (Agilent Technologies, USA) with the current sensing mode. In addition to the current maps measured by CSAFM, current-voltage curves were acquired by setting the probe tip in contact with the passive films at different locations in ferrite and austenite phases of the current maps, which were done at least 10 times at different locations of the specimen and representative curves were picked out. The equipment was located in a clean room at a constant temperature of 25 °C and relative humidity of about 25%. The probes used in MFM measurements were Bruker magnetic probes (MESP) with force constant of 2.8 N/m, while the probes in CSAFM measurements were AppNano conductive Pt-coated silicon tips with a force constant of 2.8 N/m and a tip radius of 40 nm.

The chemical composition of the passive films formed before and after hydrogen charging was investigated with XPS with a monochromatic Al Ka radiation source and a pass energy of 25 eV. The depth profiling was performed using an Ar^+^ gun with a beam energy of 3 kV and a beam current of 1 μA. The curve fitting was performed using the commercial software XPS Peak, version 4.1, which contains the Shirley background subtraction and Gaussian-Lorentzian tail functions, to achieve better spectra fitting.
